# Combined genome-wide association studies and expression quantitative trait locus analysis uncovers a genetic regulatory network of floral organ number in a tree peony (*Paeonia suffruticosa* Andrews) breeding population

**DOI:** 10.1093/hr/uhad110

**Published:** 2023-07-05

**Authors:** Liping Peng, Yang Li, Wanqing Tan, Shangwei Wu, Qing Hao, Ningning Tong, Zhanying Wang, Zheng’an Liu, Qingyan Shu

**Affiliations:** Key Laboratory of Plant Resources, Institute of Botany, the Chinese Academy of Sciences, Beijing 100093, China; China National Botanical Garden, Beijing 100093, China; Key Laboratory of Plant Resources, Institute of Botany, the Chinese Academy of Sciences, Beijing 100093, China; China National Botanical Garden, Beijing 100093, China; Key Laboratory of Plant Resources, Institute of Botany, the Chinese Academy of Sciences, Beijing 100093, China; China National Botanical Garden, Beijing 100093, China; College of Life Science, University of Chinese Academy of Sciences, Beijing 100049, China; Key Laboratory of Plant Resources, Institute of Botany, the Chinese Academy of Sciences, Beijing 100093, China; China National Botanical Garden, Beijing 100093, China; College of Life Science, University of Chinese Academy of Sciences, Beijing 100049, China; College of Landscape Architecture and Forestry, Qingdao Agricultural University, Qingdao 266109, China; Key Laboratory of Plant Resources, Institute of Botany, the Chinese Academy of Sciences, Beijing 100093, China; China National Botanical Garden, Beijing 100093, China; Peony Research Institute, Luoyang Academy of Agricultural and Forestry Sciences, Luoyang 471000, China; Key Laboratory of Plant Resources, Institute of Botany, the Chinese Academy of Sciences, Beijing 100093, China; China National Botanical Garden, Beijing 100093, China; College of Life Science, University of Chinese Academy of Sciences, Beijing 100049, China; Key Laboratory of Plant Resources, Institute of Botany, the Chinese Academy of Sciences, Beijing 100093, China; China National Botanical Garden, Beijing 100093, China; College of Life Science, University of Chinese Academy of Sciences, Beijing 100049, China

## Abstract

Great progress has been made in our understanding of floral organ identity determination and its regulatory network in many species; however, the quantitative genetic basis of floral organ number variation is far less well understood for species-specific traits from the perspective of population variation. Here, using a tree peony (*Paeonia suffruticosa* Andrews, Paeoniaceae) cultivar population as a model, the phenotypic polymorphism and genetic variation based on genome-wide association studies (GWAS) and expression quantitative trait locus (eQTL) analysis were analyzed. Based on 24 phenotypic traits of 271 representative cultivars, the transcript profiles of 119 cultivars were obtained, which indicated abundant genetic variation in tree peony. In total, 86 GWAS-related *cis*-eQTLs and 3188 *trans*-eQTL gene pairs were found to be associated with the numbers of petals, stamens, and carpels. In addition, 19 floral organ number-related hub genes with 121 *cis*-eQTLs were obtained by weighted gene co-expression network analysis, among which five hub genes belonging to the ABCE genes of the MADS-box family and their spatial–temporal co-expression and regulatory network were constructed. These results not only help our understanding of the genetic basis of floral organ number variation during domestication, but also pave the way to studying the quantitative genetics and evolution of flower organ number and their regulatory network within populations.

## Introduction

In nature, the angiosperms have shown an infinite variety of floral forms during evolution. Flowers, as reproductive and composite structures, are typically composed of four whorls of organs, including sepals, petals, stamens, and carpels, with homeotic transformations also happening among them [1]. For humans, knowledge of floral organs is one of the keys to productive success during domestication and breeding. Understanding the domestication process and assessing the available genotypic variation in floral organs are critical to species formation and further breeding. Nowadays, more research is focused on floral organ identity and its regulatory network in terms of their evolution and development [2], which are depicted as underlain by a classical ABCE model and its derivatives in dicotyledons and monocots [3]. The identities of the four whorls of organs are generated sequentially by A-class genes [*AP1*/*2* (*APTEALA1*/*2*)] for sepals, A-class and B-class genes [*AP3* and *PI* (*PISTILLATA*)] for petals, B-class and C-class genes [*AG* (*AGAMOUS*)] for stamens, and class C genes for carpels, while E-class genes [*SEP1–4* (*SEPALLATA 1*–*4*)] in combination with the above genes underlie all four whorls of organs [4–7]. Due to huge variation in floral organ number in diverse species or cultivars, although great progress has been made on floral organ identity genes and their regulatory network, the study of floral organ number variation is still a hot topic and far less understood as a species-specific trait from the perspective of population variation.

In the past decade, people focused on several regulatory genes or their interaction network to explain flower development and floral organ identity through genetics [1, 8–11]. Recently, thanks to the large-scale sequencing and genome assembly of more than 400 species of plants [3], understanding of phenotypic polymorphism and genetic variation based on genome-wide association studies (GWAS) has been enhanced. Expression quantitative trait locus (eQTL) analysis, first proposed by Jansen and Nap [12], has become a widely used tool for identifying genetic variants that affect gene regulation [13,14] in many traits except floral organ number variation in plants, including *Arabidopsis*, tomato, maize, and rice [15–18], and could help to clarify the links between genotype and phenotype and shed light on the effects of associated variants on gene expression [19]. However, to our best knowledge, the eQTLs for floral organ number variation remain unclear, although they are very important in respect of evolution, adaptation, domestication, and breeding. Due to species-specific diversity, to resolve the question of how the variation happens and its mechanism, there is a great need to use quantitative means, such as a population genetics approach, to reach a full understanding of gene expression and phenotype variation, especially with regard to floral organ numbers in species not limited to graminaceous or model plants.

Paeoniaceae includes only a single genus (with two subgenera) and its specific position among the angiosperms is in the Saxifragales, which is closely related to Vitales as a sister group of Rosides [20, 21]. It was suggested that Paeoniaceae and Crassulaceae diverged before 109 million years ago (Mya) (102–120.8 Mya), or 110 Mya by genome evolution or *rbcL* sequence analysis [21–23]. Tree peony (*Paeonia suffruticosa* Andrews) is a woody shrub belonging to the *Paeonia* subgenus *Moutan* in the Paeoniaceae family; it is one of the world’s most precious flowers due to its high ornamental value and economic significance [24–26]. Its flower develops in an arranged specific structure from leaf to bract; from the outer to the inner part are the sepals, petals, stamens, and carpels. The stamens are numerous and the separate carpels have free nuclei during mitosis in embryo development, and in this they resemble gymnosperms [27–29] and develop into aggregated follicle. Therefore, the floral organ of the tree peony is a unique trait to understand the species formation and systematic position of Paeoniaceae. Furthermore, the large flower with various forms and colors, known as the ‘king of flowers’, symbolizes happiness, wealth, and prosperity in China, where there has been a long history of cultivation over 1600 years [25]. Because of long-term domestication and cultivation in combination with natural or artificial selection, there are >2000 cultivars worldwide, of which more than 1500 exist in China [24]. It has a small number of chromosomes (2*n* = 10), but there is a large genome, of >12 Gb [20, 21], which makes it genetically complicated. It is noteworthy that, due to the interest in petal number, there have been attempts to breed cultivars with just sepals and petals without carpels and stamens, and during the long history of breeding, the numbers of petals, stamens, and carpels have demonstrated large variation with diversity of morphology and flower form, which are considered to be the main traits for cultivar classification [24]. Unexpectedly, the tree peony has become an intriguing model for research on the mechanisms of flower diversification in woody plants as a result of large genetic diversity and variation in floral organ number.

There are two classical ways to alter the number of floral organs: (i) enlarging/reducing the overall size of the floral meristem; and (ii) homeotic transformation of one type of floral organ into another [7]. The size of the floral meristem is related to the maintenance of stem cell homeostasis. The maintenance of floral meristem activity is mainly regulated by a complex feedback loop between CLAVATA (CLV) and WUSCHEL (WUS) in which several regulatory factors, such as HAIRY MERISTEM (HAM) [30], are involved. Mutants of *CLV* genes generally produce more floral organs while the *wus* phenotype is characterized by a reduced number of floral organs [31, 32]. Alternatively, the number of floral organs can shift when one organ type is converted into another, which is called homeotic transformation. For example, the *ETTIN*/*AUXIN RESPONSE FACTOR* (*ETT*/*ARF3*) gene, which mediates auxin signaling, resulted in the production of larger flowers with increased sepal and petal number but decreased stamen number, as well as defects in the gynecium [33, 34], while silencing *ARF18* caused decreased petal number but increased stamen number [35]. Recently, it was suggested that a petaloid stamen was the major source of increased petal number due to the restricted expression of a class C gene (*AG*), and ectopic expression of a class A gene (*AP1*) in *Paeonia* [20, 21]. In our previous studies we found large sequence differences in a class B gene (*TM6*), which may also contribute to the petal number difference [36]. Additionally, 15 potential transcription factors (TFs) involved in carpel quantitative variation were identified from the MYB, WD, RING1, and LRR families in *P. rockii* [37], which indicated that there is an extremely complex process of transcriptional regulation. From a theoretical standpoint, it is easier to comprehend how flower organ number can vary in tree peony when we are aware of these processes in other species. However, considering the large diversity within tree peony cultivars, the eQTLs and genetic regulatory network underlying floral organ number variation within large cultivar groups are still less understood.

In this study, based on quantitative traits of floral organ number and transcript profiles of tree peony cultivation groups, we combined GWAS and eQTLs to explore the genetic basis of petal, stamen, and carpel number variation. This study will illuminate our understanding of the genetic basis underlying floral organ number variation during domestication from a population perspective. Meanwhile, it paves a new way to understand flower type diversity during species formation and evolution, as well as crop productivity control as an aspect of agriculture.

## Results

### Phenotypic variation and global gene expression profiling

**Figure 1:**
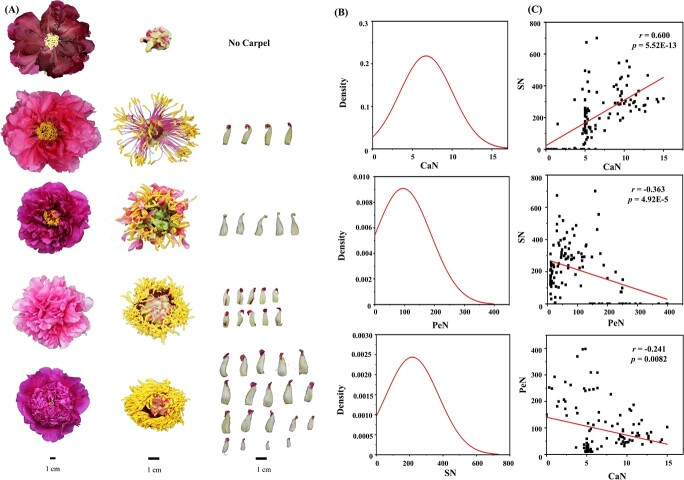
Overview of the CaN, PeN, and SN assessed in this study. (A) Representative types of floral organs. (B) Distribution of CaN, PeN, and SN of 119 cultivars. (C) Pairwise correlation between CaN, PeN, and SN.

Phenotypic variation was used as a resource for germplasm diversity, and in order to dissect the range of variation among tree peony cultivars, 24 traits of 271 representative cultivars were investigated (Supplementary Data Table S1). These traits basically demonstrated a normal distribution (Supplementary Data Fig. S2) and the coefficient of variation (CV) ranged from 9.89 to 94.77%, with a mean value of 28.67%. Petal number (PeN) had the largest CV (94.77%), followed by stamen number (SN, 65.56%) and carpel number (CaN, 40.04%) (Supplementary Data Tables S2 and S4; Fig. 1). Considering the influence on yield and ornamental values of PeN, SN, and CaN, 119 cultivars were selected for further analysis, among which the floral organ number varied from 9.67 to 398 (PeN), 0 to 700.5 (SN), and 0 to 15 (CaN) (Fig. 1B, Supplementary Data Tables S2 and S4). Further analysis indicated that PeN was negatively correlated with SN (*r* = −0.363, *P* = 4.92E−5) and CaN (*r* = −0.241, *P* = 0.0082), but SN was positively correlated with CaN (*r* = 0.600, *P* = 5.52E−13) (Fig. 1C).

Based on the full-length transcriptome of tree peony previously generated by PacBio sequencing, a total of 86 894 494 bp of 61 886 full-length transcripts with an average length of 1404 bp were obtained and used as reference sequences, among which 54 454 open reading frames (ORFs) were assembled (Supplementary Data Fig. S3A). Then, the global gene expression profiles for 119 cultivars were analyzed using Illumina HiSeq 2000, and an average of >35 445 047 high-quality reads were obtained, which were aligned to the full-length transcriptome, with an average mapping rate of 91.58% (Supplementary Data Table S5). The TPM (transcripts per kilobase of exon model per million mapped reads) value of a total of 52 280 genes from 49 615 to 50 836 in 119 cultivars was determined, accounting for 96.01% of the annotated genes (Supplementary Data Table S5).

After single-nucleotide polymorphism (SNP) and InDel calling, 540 450 SNPs/InDels were detected with a mean SNP density of 8.73 SNPs per transcript. We found that 57.78, 3.93, 19.90, and 18.38% of total SNPs were located within exons, intergenic regions, the 3′UTR, and the 5′UTR, respectively (Supplementary Data Fig. S3B). After filtering, 407 561 high-confidence SNPs (missing data <50%, minor allele frequency >5%) were identified and used for subsequent analyses.

### Relationships between transcript levels, kinship, and phenotypes

**Figure 2:**
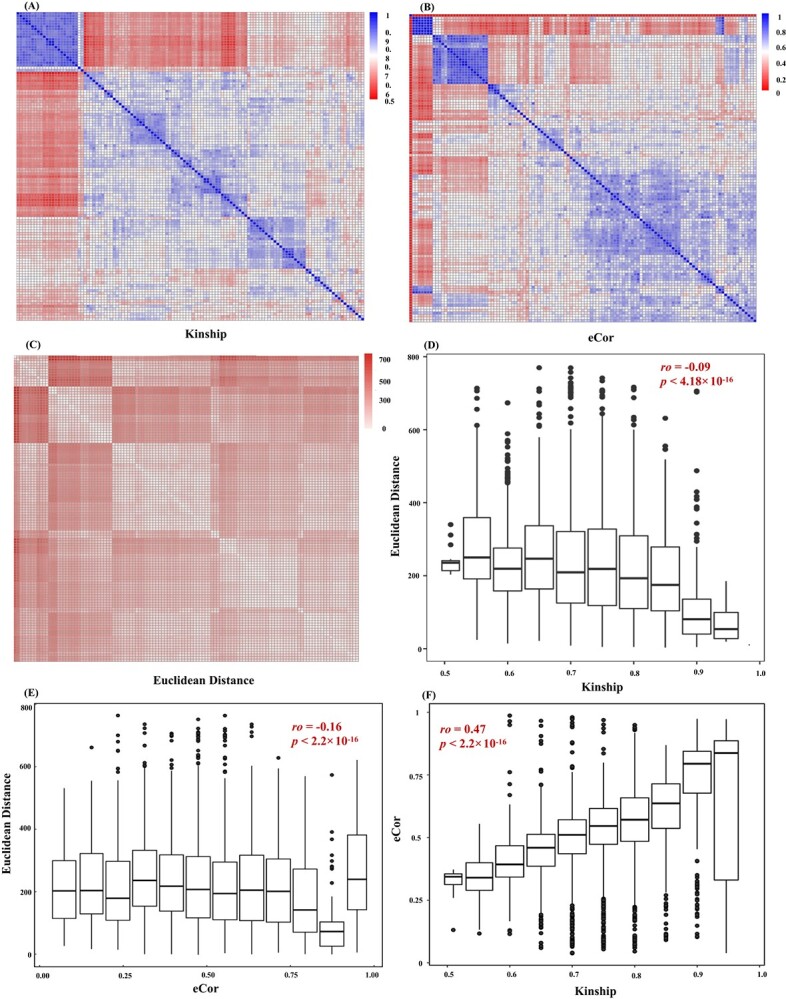
Relationships between cultivars based on transcript levels, kinship, and Euclidean distance. (A–C) Relationships between cultivars based on kinship (A), eCor (B), and Euclidean distance (C). (D, E) Relationships between Euclidean distance and kinship (D) and eCor (E). (F) Relationship between kinship based on SNPs and eCor (Pearson’s correlation coefficient).

Based on the high-confidence SNPs, we calculated the kinship between the 119 cultivars, and in order to further discriminate similar transcript levels between cultivars, the expression correlations (eCor) between all pairs of cultivars were obtained using Pearson’s correlation coefficient. Heat maps were made for kinship and the eCor matrix, and the genetic relationship values were mainly from 0.49 to 0.98 (Supplementary Data Table S6, Fig. 2A) and from 0.04 to 0.99 (mean 0.52), respectively (Supplementary Data Table S7, Fig. 2B). To understand the correlation between genetic data and phenotypic variation, the correlation between kinship or eCor and Euclidean distance (Supplementary Data Table S8, Fig. 2C) was analyzed. Both kinship (*ρ* = −0.09, *P* < 4.18 × 10^−16^) (Fig. 2D) and eCor (*ρ* = −0.16, *P* < 2.2 × 10^−16^) (Fig. 2E) were significantly negatively correlated with the Euclidean distance. In addition, cultivars with similar transcriptome profiles were also genetically similar, as there was a significant correlation between values of eCor and the kinship matrix (*ρ* = 0.47, *P* < 2.2 × 10^−16^) (Fig. 2F). Taken together, these results suggest that transcript levels may be as informative as kinship but explain phenotypic variation from different aspects.

### Population structure and genetic variation

**Figure 3:**
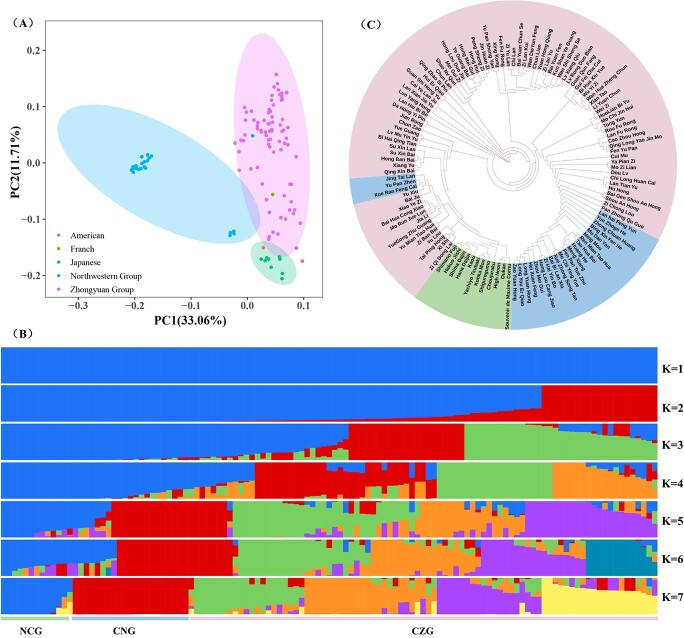
Genetic structure analysis of tree peony cultivars. (A) PCA plot, which shows that all genotypes can be divided into three clusters. (B) Population structure distribution at *K* values from 1 to 7. (C) Phylogenetic tree based on 407 561 SNPs. Chinese Zhongyuan cultivation group (CZG), Chinese Northwest cultivation group, (NCG) and non-Chinese cultivation group (NCG) are shown in pink, blue, and green in (A) and (C).

Meanwhile, 119 cultivars from the Chinese Zhongyuan cultivation group (CZG; 81), the Chinese Northwest cultivation group (CNG; 26), and the non-Chinese cultivation group (NCG; 10 Japanese cultivars, 1 French cultivar, and 1 American cultivar) were clustered in three classes containing 10, 26, and 83 cultivars, respectively (Fig. 3A), which roughly corresponded to the classification of cultivation groups and their origins. The 119 cultivars were further classified into six clusters (*K* = 7) based on population structure analysis, with similar clusters shown by principal component analysis (PCA), except that CZG was further divided into four clusters (Fig. 3B). Similar results were obtained by phylogenetic analysis (Fig. 3C), indicating the different genetic distances among these three groups. Furthermore, it indicated that the average nucleotide diversity (π) and polymorphism [Waterson’s theta, (θ_W_)] of CNG (π = 3.50 × 10^−4^, θ_W_ = 5.00 × 10^−4^) and NCG (π = 4.02 × 10^−4^, θ_W_ = 4.58 × 10^−4^) were lower than those of CZG (π = 4.72 × 10^−4^, θ_W_ = 6.26 × 10^−4^) (Table 1). The *F*_ST_ value was lowest (0.149) between NCG and CNG, and highest between CNG and CZG (0.405). These results confirmed that our panel could capture abundant genetic variations of tree peony cultivars.

**Table 1:** Polymorphism and variation in different tree peony groups.

**Parameter**		**All**	**Group**		
			**CZG**	**CNG**	**NCG**
No. of cultivars		119	82	25	12
Variation	π	5.84 × 10^−4^	4.72 × 10^−4^	3.50 × 10^−4^	4.02 × 10^−4^
	θ_W_	6.67 × 10^−4^	6.26 × 10^−4^	5.00 × 10^−4^	4.58 × 10^−4^
Population divergence			CZG and CNG	CZG and NCG	CNG and NCG
	*F*_ST_		0.303	0.405	0.149

CZG, Chinese Zhongyuan cultivation group; CNG, Chinese Northwest cultivation group, NCG, non-Chinese cultivation group; π and θ_W_, index of nucleotide diversity; *F*_ST_ values were calculated for each 10-kb sliding window with a step size of 5 kb for each SNP among the populations.

### GWAS and eQTLs reveal allelic variants of genes associated with floral organ numbers

**Figure 4:**
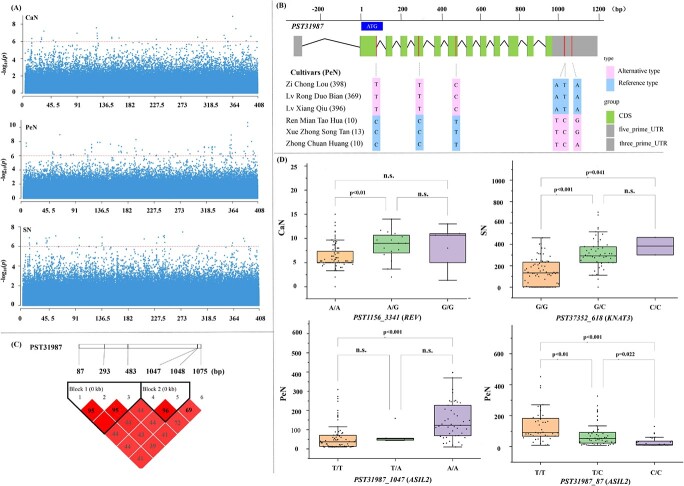
GWAS of CaN, PeN, SN, and phenotypic variation caused by SNPs. (A) Manhattan plots of associations between SNPs and CaN, PeN, and SN. (B) Allelic information on sequence variants in *PST31987* (*ASIL2*) for six cultivars. The number in brackets after each cultivar name indicates average PeN as a whole number. (C) Pairwise linkage disequilibrium between associated SNPs and SNPs within *PST31987* (*ASIL2*). Numbers in the red boxes are probabilities of linkage. (D) Divergence of PeN, CaN, and SN between different alleles of the SNP. The *x*-axis indicates the different alleles of the lead SNPs in *PST1156* (*REV*), *PST37352* (*KNAT3*), and *PST31987* (*ASIL2*), and the *y*-axis represents the different number of the carpel, stamen, and petal.

CaN, PeN, and SN are the vital traits associated with tree peony domestication and breeding, and are the references for classification. We performed GWAS for CaN, PeN, and SN, and 255 significant SNP–trait associations were identified, which were distributed among 176 transcripts. Thirty-four, 141 and 85 SNPs were significantly (*P* < 0.01) associated with CaN, PeN, and SN, respectively in three different environments (2020, 2021, and average) (Fig. 4A, Supplementary Data Fig. S4A, Supplementary Data Table S9). Furthermore, a total of 407 561 SNPs (minor allele frequency >5% and missing rate <30%) and the expression levels of 51 789 unigenes were used for eQTL mapping. We detected 181 952 *cis*-eQTLs located in 36 174 genes, and 24 458 080 *trans*-eQTLs for 46 989 genes, with an average of 5.03 *cis*-eQTLs and 520.51 *trans*-eQTLs per gene, among which a total of 86 GWAS-related *cis*-eQTLs (Supplementary Data Fig. S4B and C, Supplementary Data Table S10) and 3188 *trans*-eQTL gene pairs (Supplementary Data Table S11) were found to be associated with three traits at a false discovery rate (FDR) of <0.05.

Interestingly, six SNPs of PST31987, encoding homology protein of ASIL2 (*Arabidopsis* 6b-interacting protein 1-like 2), were significantly correlated with PeN (Supplementary Data Table S9, Fig. 4B). Further linkage disequilibrium analysis for these SNPs showed that there were two block regions, one containing three loci (87, 293, and 483 bp) and the other containing two loci (1047 and 1048 bp) (Fig. 4C). We selected two representative SNP loci (87 and 1047 bp) in these two block regions and found that the variation of the two loci was correlated with the divergence of PeN. Among the GWAS-related *cis*-eQTLs, we found that two different genotypes of PST1156 that were annotated as *REVOLUTA* (*REV*) corresponded to differential CaN (Fig. 4D). It is noteworthy that an SNP of PST37352, annotated as *KNOTTED1-LIKE HOMEOBOX GENE 3* (*KNAT3*), was detected for the variation of SN (Fig. 4D). This suggests that genetic variants generated in this study could result in the shaping of phenotypic differences.

### eQTLs for floral organ number-related modules and the co-expressed network

**Figure 5:**
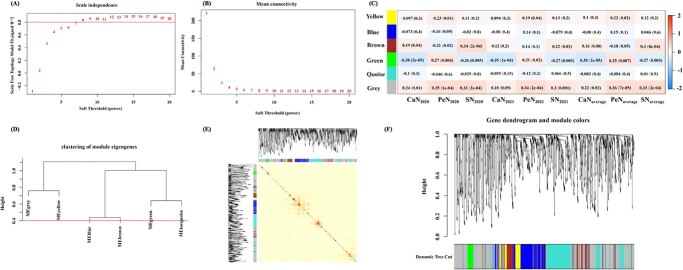
Gene co-expression networks analyzed by WGCNA. (A) Scale independence. (B) Mean connectivity. (A) and (B) were used to determine the soft threshold (*β* value) at which the gene co-expression network became close to scale-free. (C) Heat map of correlations between CaN, PeN, and SN and the detected modules in three environments. Values within the heat map indicate *P* values (in parentheses) and *r* values (correlation coefficients). (D) Eigengene cluster tree. The modules were merged below the red line (at 0.4) in the next step. (E) TOM map. Rows and columns indicate individual genes, and red and deep yellow colors indicate high topological overlap. (F) Gene cluster tree (above) and different modules (below).

The expression profiles of 52 280 genes in the 119 cultivars were further analyzed using Gene Ontology (GO) analysis. This showed that 1062 genes were enriched in the processes putatively related to flower development (GO:0009908). In order to see whether 176 transcripts identified by GWAS associated with floral organs number are co-expressed with the 1062 flower development-related genes, we further conducted weighted gene co-expression network analysis (WGCNA) and six modules were obtained (Fig. 5A–C) at the height threshold of 0.4 (Fig. 5D). This indicated that three modules including MEGrey, MEBrown, and MEGreen were relatively highly correlated with floral organ numbers, consisting of 455, 122, and 40 genes, respectively, which demonstrated similar trends in the three environments, i.e. in the average environment CaN (*r* = −0.38, *P* = 2E-5) and SN (*r* = −0.27, *P* = 0.003) were negatively correlated with MEGreen, while positive correlations of PeN (*r* = 0.36, *P* = 7E-5) and SN (*r* = 0.33, *P* = 2E-4) with MEGrey and of SN (*r* = 0.3, *P* = 8E-4) with MEBrown were observed.

**Figure 6:**
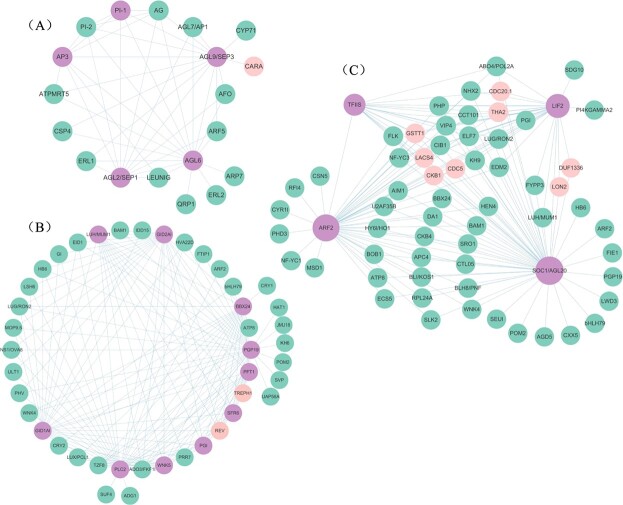
Co-expression network of 19 hub genes from MEgreen, MEgrey, and MEbrown related to floral organ numbers. Five, four and ten hub genes with co-expressed node connectivity were identified in (A) MEgreen, (B) MEbrown, and (C) MEgrey, respectively. Hub genes are shown in purple and GWAS-related *cis*-eQTL distributed genes are shown in pink.

A total of 3066, 721, and 96 *cis*-eQTLs regulating 356, 122, and 15 genes were identified in MEGrey, MEBrown, and MEGreen, respectively, and 53 GWAS-related *cis*-eQTLs in 36 genes were identified (Supplementary Data Table S12). Furthermore, 19 floral organ number-related hub genes [eigengene connectivity (KME) > 0.7] with 121 *cis*-eQTLs were identified (Supplementary Data Table S13). There were 5 hub genes in MEGreen, including *AGL6* (*AG-LIKE6*), *AP3*, *PI-1*, *AGL4*/*SEP2*, and *AGL9/SEP3*, 10 in MEBrown, including *PFT1* (*PHYTOCHROME AND FLOWERING TIME 1*), *PLC2* (*PHOSPHOLIPASE C 2*), *PGP19* (*P-GLYCOPROTEIN 19*), *PGI* (*PHOSPHOGLUCOSE ISOMERASE 1*), *GID1A* (*GA INSENSITIVE DWARF1A*), *SFR6*/*MED16* (*SENSITIVE TO FREEZING 6*/*MEDIATOR 16*), *LUH*/*MUM1* (*LEUNIG_HOMOLOG*/*MUCILAGE-MODIFIED 1*), *BBX24* (*B-BOX DOMAIN PROTEIN 24*), *WNK5* (*WITH NO LYSINE (K) KINASE 5*), and *GID2A* (*GA INSENSITIVE DWARF 2A*), and 4 in MEGrey, including *LIF2* (*LHP1-INTERACTING FACTOR 2*), *TFIIS* (*TRANSCRIPT ELONGATION FACTOR IIS*), *SOC1* (*SUPPRESSOR OF OVEREXPRESSION OF CO 1*)/*AGL20*, and *ARF2* (*AUXIN RESPONSE FACTOR 2*)*.* GO analysis indicated that all hub genes in MEGreen, including *AGL6*, *AP3*, *PI-1*, *AGL4*/*SEP2*, and *AGL9*/*SEP3*, belonging to the ABCE class MADS-box genes, were related to floral organ formation (GO:0048449) and floral organ morphogenesis (GO:0048444); meanwhile, *AGL6* was also related to floral whorl morphogenesis (GO:0048457), and *AGL6* and *AGL9*/*SEP3* were related to specification of floral organ number (GO:0048833). The other hub genes were also involved in the process of regulation of flower development (*PFT1*, *PGI*, *LUH*/*MUM1*, *LIF2*, *TFIIS*, *SOC1*/*AGL20*, and *ARF2*; GO:0009909), floral organ development (*GID1A*, *PLC2*, *PGP19*, and *ARF2*; GO:0048437), photoperiodism and flowering (*BBX24*, GO:0048573), and regulation of photoperiodism and flowering (*SFR6*/*MED16*; GO:2000028) (Supplementary Data Table S13). Then, we constructed the co-expression network of 19 hub genes and 15, 61, and 33 co-expressed nodes with high connectivity identified in MEGreen, MEGrey, and MEBrown, respectively (Fig. 6, Supplementary Data Table S14), among which 11 genes were regulated by GWAS-related *cis*-eQTLs.

**Figure 7:**
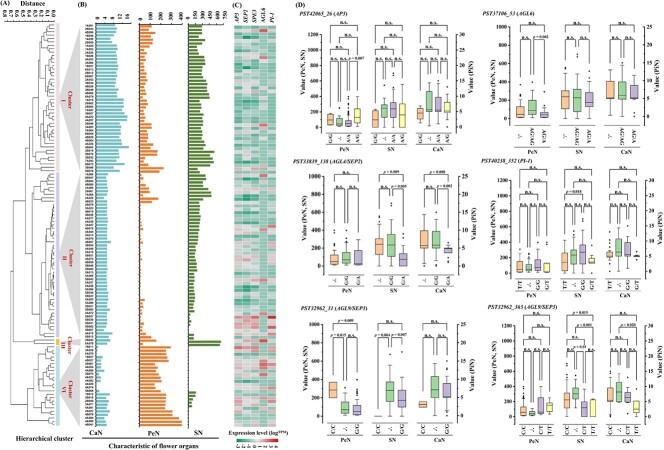
Phylogenetic clusters of floral organ number, candidate gene expression patterns, and corresponding phenotype variation among 119 cultivars. (A) Phylogeny based on Euclidean distance of CaN, PeN, and SN. (B) Clustering of floral organ number. (C) Expression patterns of five hub genes in MEGreen. Expression levels were evaluated using TPM. (D) Divergence of floral organ number between different alleles of the eQTLs.

**Figure 8:**
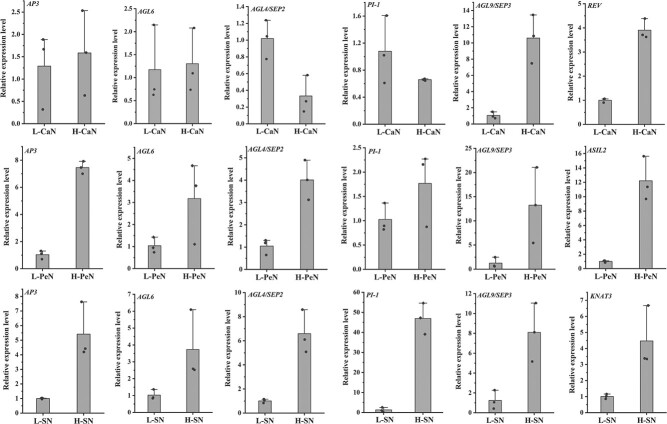
qRT–PCR validation of candidate genes associated with floral organ number. L-CaN, L-PeN, and L-SN indicate samples with smaller CaN, PeN, and SN values respectively, and H-CaN, H-PeN, and H-SN indicate samples with higher CaN, PeN, and SN values, respectively.

We further focused on five hub genes with eQTLs in MEGreen and were interested to see that their expression patterns complement the phylogenetic clusters of 119 cultivars reconstructed using Euclidean distances of CaN, PeN, and SN (Fig. 7A–C). Meanwhile, we found that the eQTLs of *AP3*, *AGL6*, and *SEP3*/*AGL9* corresponded to differential PeN; *SEP2*/*AGL4*, *SEP3*/*AGL9*, and *PI-1* were responsible for the variation of SN; and *SEP2*/*AGL4* and *SEP3*/*AGL9* resulted in the divergence of CaN (Fig. 7D, Supplementary Data Fig. S5). The relationship between transcriptional levels and floral organ number variation was further confirmed by quantitative real-time PCR (qRT–PCR) (Fig. 8).

### Spatial–temporal co-expression and regulatory network

**Figure 9:**
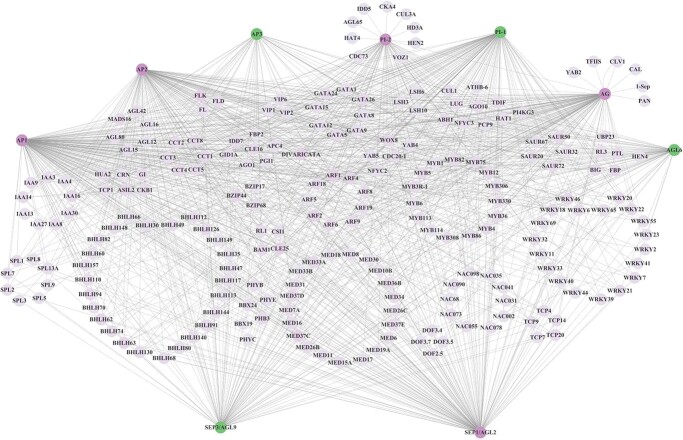
Co-expressed network of ABCE genes with high connectivity nodes. ABCE genes that affect the number of floral organs are shown in green.

**Figure 10:**
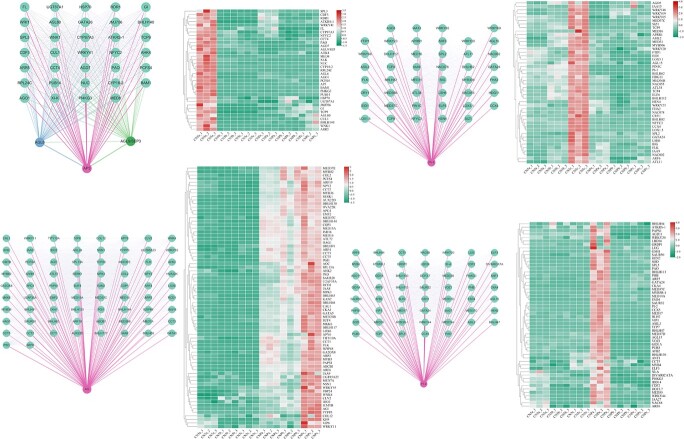
Spatial–temporal co-expression network for ABCE class MADS-box genes and their high connective nodes, which may affect the number of floral organs.

**Figure 11:**
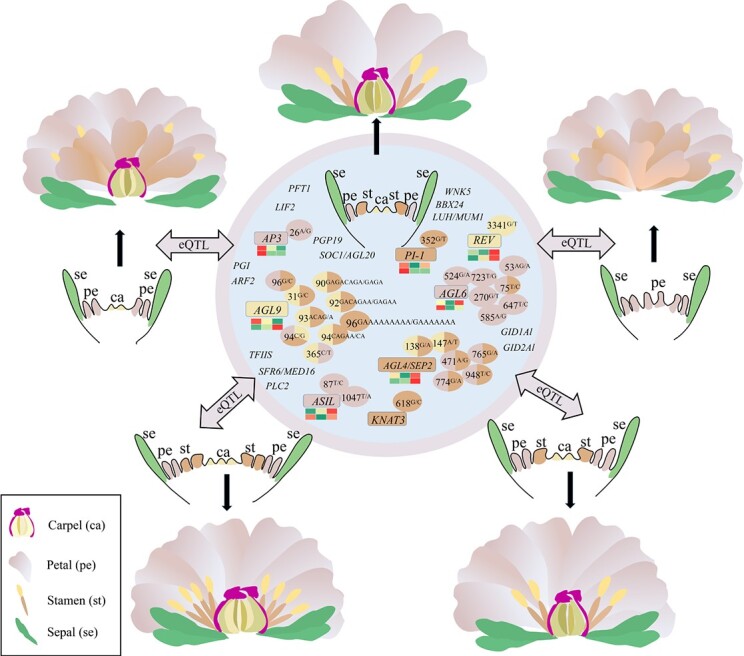
Hypothetical model of the regulatory networks for floral organ number variation in tree peony.

To further investigate the spatial–temporal expression pattern of the above five ABCE class MADS-box genes, we obtained the transcriptome profiles of flower buds at three developmental stages from two cultivars, and a total of 41 280 unigenes and 32 079 differentially expressed genes (DEGs) were identified, among which genes enriched in flower development were selected (Supplementary Data Table S15). Floral organ number is determined by many TF families, such as MADS, AP2, MYB, basic helix–loop–helix (bHLH), NAC, TCP, WRKY, bZIP, and CCT, and those involved in the auxin and cytokinin signal pathways, therefore, 2179 related DEGs were used for WGCNA. We further constructed a co-expressed network of ABCE class MADS-box genes with high connectivity nodes [topological overlap matrix (TOM) > 0.2] (Fig. 9), which corresponded to their spatial–temporal expression pattern (Fig. 10). Then, a hypothetical model of the regulatory network for the floral organ number variation in tree peony was proposed, as shown in Fig. 11

## Discussion

The evolution and domestication of plants are beneficial to species formation, adaptation, and humans’ needs in relation to food supplies, and floral organ number has been highlighted as having significant functions in recent studies [19]. Tree peony’s flower petal, stamen, and carpel numbers have presented high diversity during its domestication and breeding, which could be used as a dedicated model for dissecting floral organ number variation. As complex quantitative traits are controlled by multiple genes or QTLs, characterizing the genetic basis of floral organ number is challenging. However, considering the advantage of connecting the quantification of expression and population phenotype variation via RNA sequencing (RNA-seq), it would provide tremendous opportunities [38]. In this study, among 271 cultivars, RNA-seq data of 119 representative ones were generated, and a systematically genetic method involving integration of GWAS, eQTLs, and co-expression network was successfully used to explore the mechanism of genetic variation of floral organ number. The key genes and eQTLs that were identified will facilitate the mining of genes controlling complex traits in tree peony and the analysis of regulatory mechanisms. This study should illuminate the genetic basis underlying floral organ number variation during domestication and breeding.

GWAS is an essential means to dissect complex trait variation using large numbers of markers (typically SNPs) in many plants with genome information [39]. While, being representative of population germplasm materials, clear population structure, together with the uniformity and density of molecular markers are the major factors affecting GWAS [40]. In this study, despite the stable number of five sepals (Supplementary Data Table S1), the tree peony cultivar population showed considerable variation in the traits of PeN, SN, and CaN (CV = 94.77, 65.56 and 40.04%, respectively), demonstrating that it could be used as the representative germplasm for genetic analysis of floral organ number variation. Notably, among the four types of floral organ of tree peony, the stamen shows the maximum range of variation in number, from 0 to 700.5, which is similar to *Nigella damascena*, indicating that SN may be the determinant of flower structure [41]. In addition, since population structure is vital in GWAS, based on population structure and PCA, 119 tree peony cultivars were clustered into three groups with high-density SNPs in this study, which roughly correspond to the reported cultivar groups [24]. With the recent advances in genome information on *P. suffruticosa* [20, 21], plenty of genetic variants have been confirmed in only a few accessions, which could not explain the floral organ number diversity. Over recent decades, GWAS has been used for the genetic analysis of tree peony fatty acid biosynthesis [21], petal length, and flowering time [42], but its application to the genetic mechanism underlying floral organ number variation has remained scant. Our research obtained 255 significant SNP–trait associations for floral organ number by GWAS, which is filling the research blank.

Though GWAS is a powerful way to identify candidate associations by examining the frequency of different genotypes related to phenotypes, it is not sufficient to provide insight into biological mechanisms and define the function of the genes involved. The efficient genetic means of eQTL was explored in the latest research to understand gene expression levels and trait variation in a few plants. It is reported that eQTLs were identified for metabolite pathway specification, kernel oil variation and development, leaf development, flowering time, and height and yields in maize [17, 43–46], and starch content and diterpene antitoxin synthesis in leaves were identified in rice [18]. In horticultural plants, eQTLs related to fruit metabolite traits and pathogen response in tomato [16, 47], lignin and cellulose biosynthesis in pear fruit [48], the initiation of secondary cell wall development in cotton [49], clove shape trait in garlic [50], and growth, wood quality, and oleoresin traits in the slash pine [51] were also obtained. However, to our best knowledge, the eQTLs for floral organ number variation have remained unclear. In this study, we pioneered the integration of summary-level data from GWAS and eQTLs to reach a full understanding of gene expression and floral organ number variation. Most studies have concerned only *cis*-eQTL effects when considering genes for complex traits; however, our study also combines *cis*-SNPs (by GWAS and eQTLs) into a single predictor. Compared with individual SNPs or *cis*-eQTLs, combined *cis*-SNPs may capture heterogeneous signals better at the same time. Furthermore, our focus on predicting the genetic component of expression also avoids confusion arising from environmental differences caused by traits influencing expression [52]. Moreover, in the present study, a total of 3188 GWAS-related *trans*-eQTL gene pairs were associated with CaN, PeN, and SN. The *trans*-eQTLs, whose SNPs are located distal to the gene (>5 Mb) or on another chromosome, generally have smaller effect sizes than those of *cis*-eQTLs, and thus were neglected in the majority of studies [53]. However, *trans*-eQTLs could also be associated with complex traits, because the *trans* effect of each individual is unlikely to be suppressed by post-transcriptional compensation pools or removed from the population by negative selection [54, 55]. Integration of many weak *trans*-eQTL effects is suggested to account for most variations, demonstrating the great importance of distal effects [53]. Therefore, further research should be undertaken to investigate the *trans*-eQTLs associated with floral organ number. In conclusion, systematic and large-scale studies on *cis*- and *trans*-eQTLs of various species of tree peony with diverse flower phenotypes would provide a basis for further understanding of the effects of genetic variation on flower organ numbers and flower development.

Among these identified GWAS-related *cis*-eQTLs, one associated with CaN regulates the expression of *REV*. *REV* is a member of a small homeodomain-leucine zipper family that is necessary to promote parietal meristem growth and limit leaf and stem cell division of *A. thaliana* [56]. *SlREV* had functions similar to those of *AtREV* in establishment of organ polarity, regulation of SAM growth [56–58], and patterning of the vasculature [59]. Plants overexpressing *SlREV* displayed ectopic carpel-like structures between sepals and petals [60], and *avb1*, as a functional acquisition mutation of *IFL1*/*REV*, was related to changes in the polarity of stems, leaves, and carpels [61]. In accordance with the above studies, the present results have demonstrated that two different genotype variations of the *cis*-eQTLs in *REV* would cause the variation of CaN, which confirmed its function. Notably, besides the 86 GWAS-related *cis*-eQTLs we obtained, the total number of floral organs in a flower would be determined by many factors [62], as any type of floral organ can change in size or number during the flower developmental process from initiation, identity determination, morphogenesis, and maturation, which are regulated by many co-expressed networks. Therefore, we conducted WGCNA to further confirm whether existing co-expressed genes are regulated by GWAS-related *cis*-eQTLs and involved in flower development. It is somewhat exciting that module-trait relationships of WGCNA showing 19 floral organ number-related hub genes were co-expressed with 11 GWAS-related *cis*-eQTLs. Especially, the five genes with *cis*-eQTLs in MEGreen, including *AP3*, *AGL4*/*SEP2*, *AGL9*/*SEP3*, *AGL6*, and *PI-1* belonging to the ABCE class MADS-box genes, were related to floral organ formation and morphogenesis. It is now well established from a variety of studies that floral organs acquire their distinct identities according to the ABCE model of flower development [4]. No matter how diverse the floral organs, the flower develops through an ancient and conserved modular program [7], but the functional domains of the genetic program that specify the identity of different types of floral organs are flexible [41]. Previous research indicated that loss of function or restricted expression in the fourth whorl showed a strong indication of excessive number of petals in *Thalictrum thalictroides* [63], *Prunus lannesiana* [64], *Camellia japonica* [65], and rose [66], *etc*. However, inactivation or disruption of *AP3-3* was highly correlated with loss of petals and the shifting of the petal–stamen boundary within the Ranunculaceae family [41, 67]. In rose, silencing *RhAG* could significantly increase petal number resulting from petaloid stamens, while silencing of *RhHDA6* increased H3K9/K14 acetylation levels at the adjacent RhARF18-binding site of the *RhAG* promoter and reduced petal number [68, 69]. The above limited researches illuminate that the expression level of ABCE class genes is very important in respect of floral organ number variation. We found that the *cis*-eQTLs of *AP3*, *AGL6*, *SEP3*/*AGL9*, *AGL4*/*SEP2*, and *PI-1* corresponded to differential PeN, SN, and CaN, which indicated that these *cis*-eQTLs regulate the expression of genes that might cause the change in floral organ number [7]. To our best knowledge, the eQTLs of ABCE class genes have not been identified in other plants; our study will provide new insight on flower development and genetic regulation of floral organ number variation during plant domestication and evolution in angiosperm plants.

## Materials and methods

### Plant materials and phenotypic trait determination

A total of 271 cultivars of tree peony (*P. suffruticosa*) were collected at Luoyang Academy of Agricultural and Forestry Sciences, Henan Province (112°28′E, 34°28′N), including most of the widely cultivated ones from the Chinese Zhongyuan cultivation group (CZG), the Chinese Northwest cultivation group (CNG), and the non-Chinese cultivation group (NCG), and 24 phenotypic traits with at least three repetitions for each cultivar were investigated in 2020 and 2021 (Supplementary Data Fig. S1, Supplementary Data Tables S1 and S2). For CaN, PeN, and SN, three flowers were measured for each plant, with three plants for each cultivar. The three developmental stages of flower buds were same as in previous research [35, 70]: sepals and a few petal primordia appeared at stage 1 (S1); stamen primordia initiated at S2; and carpel primordia initiated at S3. Among 271 cultivars, the flower buds (equal weight of mixed S1–S3 flower buds) of 119 selected cultivars with large phenotypic variation were used for RNA-seq (Supplementary Data Table S2). Moreover, we randomly selected the flower buds from S1 to S3 of two cultivars with significant phenotypic differences for RNA-seq.

### RNA-seq, function annotations, and gene expression analysis

Qualified RNA samples were collected for construction of cDNA libraries and then sequencing on the Illumina HiSeq 4000 platform utilizing the PE150 sequencing strategy. Using Iso-Seq3 of SMRT analysis software, which carries out pre-processing for SMRT reads, *de novo* discovery of isoforms by CCS (circular consensus calling), sequencing primers and barcode removal, hierarchical clustering and iterative merging, and polishing [71], the full-length transcript sequences of flower buds, roots, and stem of *P. ostii* ‘Fengdan’ were processed. Finally, the clustered transcripts were merged into complete consensus sequences, and redundant sequences were removed with CD-HIT [72]. Potential ORFs were predicted by TransDecoder v5.6.0 (http://transdecoder.github.io). All qualified transcripts were subjected to functional annotation by searching against the GO, COG, KOG, Pfam, and KEGG databases [73].

All clean reads of each sample were mapped to the reference transcriptome generated by PacBio sequencing using Bowtie software [73]. The expression levels of each transcript in each sample were quantified by estimating the TPM values using RSEM software [74].

### SNP calling and genotyping

SNPs were identified through the SNP calling process in GATK (Genome Analysis Toolkit) [75], and all variants were annotated using SnpEff v4.5 [76]. Valid SNPs were obtained according to the following criteria: (i) the sequencing depth of the SNP was ≥2.0, (2) the minor allele frequency of the SNP was ≥0.05, and (iii) the deletion rate of the SNP in the population was ≤0.3. SNPs can be classified into homozygous SNPs (only one allele) and heterozygous SNPs (two or more alleles) according to the number of alleles per SNP site.

### Genetic diversity, population structure, and phylogenetic analyses

Genetic diversity indices (π and θw) and genetic differentiation (*F*_ST_) were calculated using VCFtools [77] with a sliding window of 10 000 bp and a step size of 5000 bp. θ_W_ and π were calculated based on the number of different loci in all individual sequences in the population, and the mean number of differential sites of any two individual sequences in the population, respectively. PCA was performed using R software v4.1.3 (the following is the same). The population structure was analyzed based on FastStructure [78] to figure out the total degree of genetic variation and its distribution pattern among the tree peony breeding populations, and the function Choosek.py was used to judge the best *K* value. In order to analyze the relationships between 119 cultivars, phylogenetic tree was constructed using MEGA10 and further visualized, annotated, and managed by Itol (https://itol.embl.de). The kinship matrix was calculated using EMMAX-kin and visualized by R software based on the effective SNPs. The correlation between eCor and kinship was calculated utilizing the Spearman’s rank coefficient (*r*) in R software.

### SNP association for phenotypic traits

The EMMAX model was used for association analysis based on the 407 561 filtered SNPs, and the *P*-value threshold was determined as 1/valid SNP loci (Bonferroni correction) to determine the SNP significance [79]. The threshold value of *P* in this study should have been 2.45 × 10^−6^; however, we chose a more stringent one of 1 × 10^−6^ to avoid false-positive RNA-seq variants. Deviation of the *P* value from expectation was evaluated utilizing Manhattan and Quantile–Quantile (QQ) plots, visualized using R software.

### Identification of eQTLs

The Matrix eQTL [80] was used for the identification of *cis*-eQTLs with *P* value <0.05. To identify *trans*-eQTLs of genes that affected the number of floral organs, gene expression levels were analyzed. Genes with TPM values >0.5 in 90% of the samples were chosen, and at least 2-fold difference between two samples represented the 5th and 95th percentiles of sorted expression levels, respectively [49], and 51 789 effective genes were screened. Then, the association analysis was conducted using EMMAX [81] for *trans*-eQTL gene pair (*P* < 1 × 10^−6^) identification with the expression levels of these genes as phenotypes, 407 561 SNPs as genotype, and PCA and kinship as covariant, respectively.

### Co-expression analysis

The package WGCNA v1.7.3 in R was used for the co-expression network analysis. Then, the ‘adjacency’ function in WGCNA was used for calculating the adjacencies with a soft-thresholding power of 6. To identify modules of co-expressed genes, the function ‘TOMsimilarity’ was used for calculating the topological overlap matrix (TOM) between all genes. Then, for average linkage hierarchical clustering, the TOM values were used as the input. The clustered gene tree was cut into different modules by utilizing the dynamic shearing method (value = 0.4), and 30 genes was the minimum number in the module. The module eigengene (ME) was summarized for each module, and modules at 0.4 (below the red line) would be merged in the next step, which indicated that their correlation was >0.8. Then, the KME value was calculated for the identification of hub genes in modules. Association analysis between merged ME and phenotypic traits was conducted. The co-expression relationships between key genes and their high node connectivity were obtained from TOM at the threshold of 0.01. The floral organ number genes’ co-expression networks composed of the credible connections were visualized utilizing Cytoscape v3.6.0 [82].

### qRT–PCR analysis

Three samples with the largest and smallest numbers of carpels, petals, and stamens were selected for quantitative analysis. A total of eight genes were selected for qRT–PCR analysis and the *ß*-*Tublin* (*ß*-*Tub*) gene was used as the internal reference for normalization [83]. The results were visualized by Origin 2022. The primers are listed in Supplementary Data Table S3.

### Statistical analyses

Correlation and normality tests on phenotypes, phylogenetic cluster construction based on Euclidean distance, and significance analysis were performed using Origin 2022.

## Acknowledgements

We would like to thank Miss Fan Kong, Mr Xiao Zhang, and Miss Miaomiao Song from the Institute of Botany, Chinese Academy of Sciences, for helping to collect flower buds and investigate phenotypical traits. We appreciate Miss Xiaohui Wang and Mr Kun Han from Luoyang Academy of Agricultural and Forestry Sciences for collecting phenotypical trait data. We thank Dr Yuannian Jiao for kind suggestions. This work was supported by the National Natural Science Foundation of China (Grant No. 32072065).

## Author contributions

S.Q. and L.Z. conceived and supervised the study. P.L., S.Q., L.Y., T.W., L.Z., and W.Z. collected phenotype traits. L.Y., P.L., W.S., and T.N. conducted the experiments. P.L. and S.Q. wrote the manuscript. P.L., L.Y., T.W., and H.Q. performed data analyses. S.Q. and L.Y. revised the manuscript. All the authors read and approved the final manuscript.

## Data availability

The data that support the findings of this study are available from the corresponding author upon reasonable request.

## Conflict of interest

The authors declare that they have no conflict of interest.

## Supplementary data

Supplementary data are available at *Horticulture Research* online.

## Supplementary Material

Web_Material_uhad110
