# Supplementary material for: Combined genome-wide association studies and expression quantitative trait locus analysis uncovers a genetic regulatory network of floral organ number in a tree peony (Paeonia suffruticosa Andrews) breeding population
Source: Hortic Res. 2023 Jul 5;10(7):uhad110. doi: 10.1093/hr/uhad110 (PMC10419549; doi:10.1093/hr/uhad110)
Supplement: Web_Material_uhad110 [file web_material_uhad110.zip › Supplemental Figures.docx]

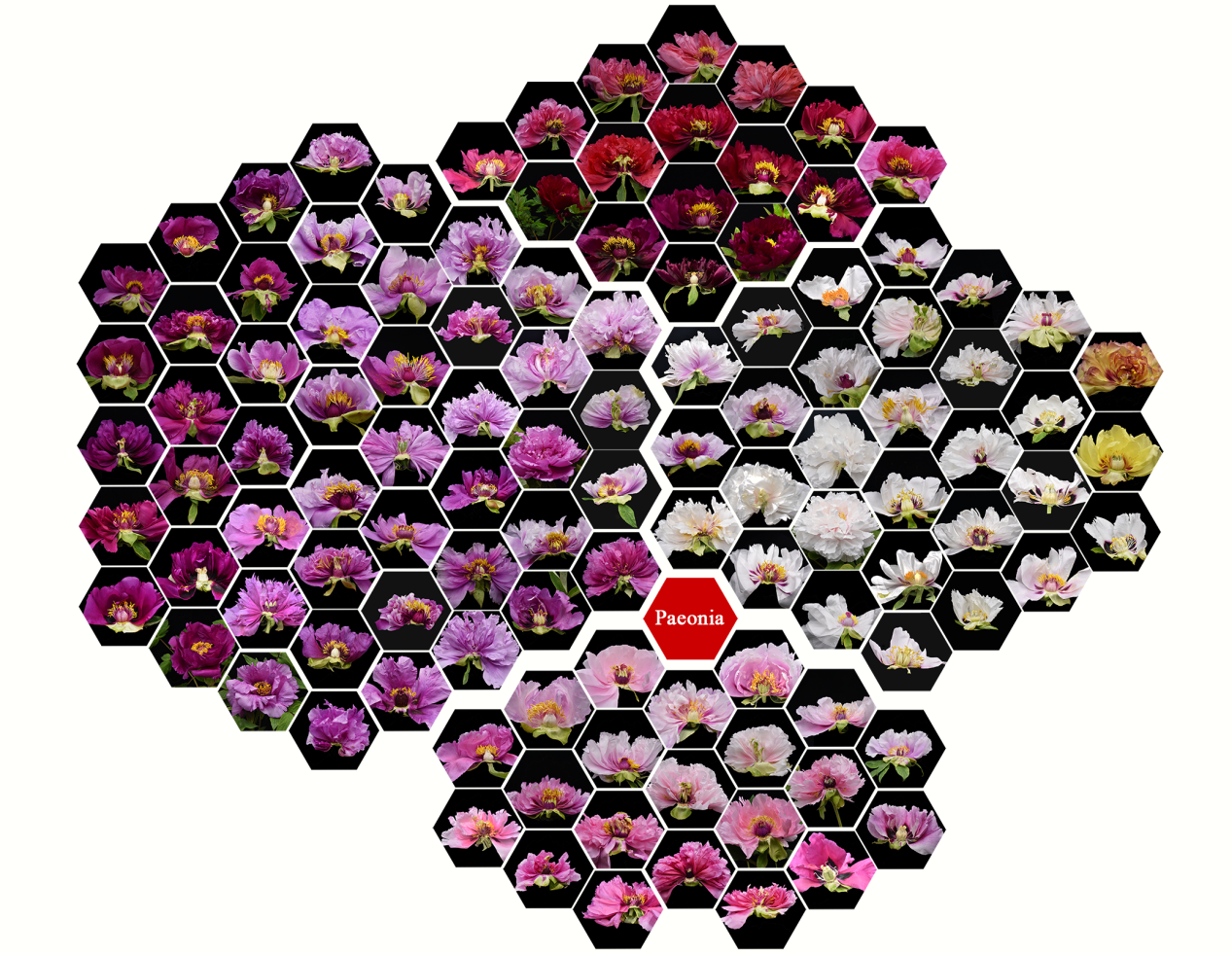


**Fig. S1** Flowers of partial tree peony cultivars.


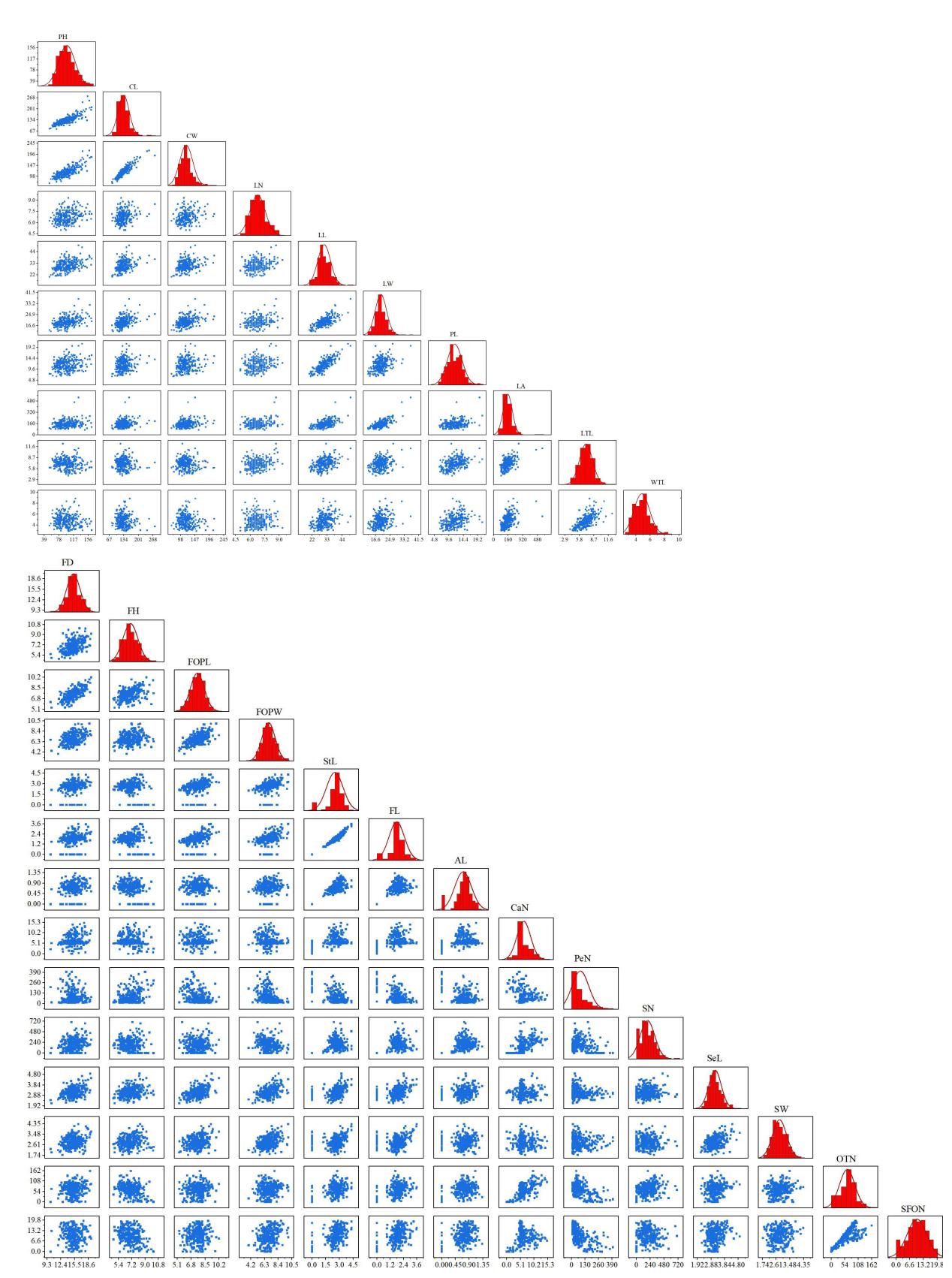


**Fig. S2** Diagram of phenotypic normality test and correlation analysis.


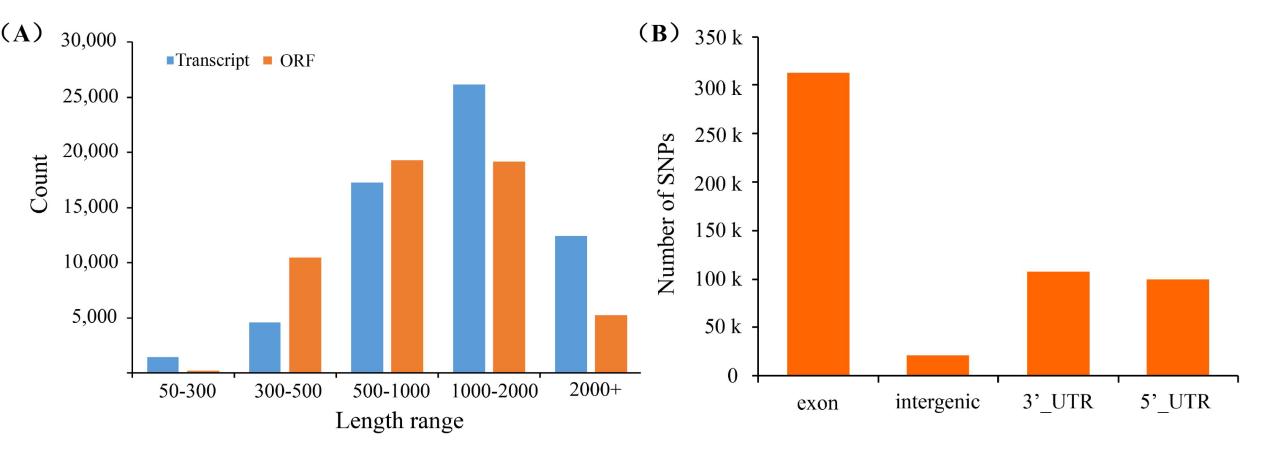


**Fig. S3** Count number of transcript and ORF in the corresponding length range in full-length transcript, and the SNP location in the RNA-seq dataset. (A) The count number of transcript and ORF with different length range in the full-length transcript dataset. (B) The number of SNPs in each region: exon, intergenic, 3' UTR and 5' UTR.


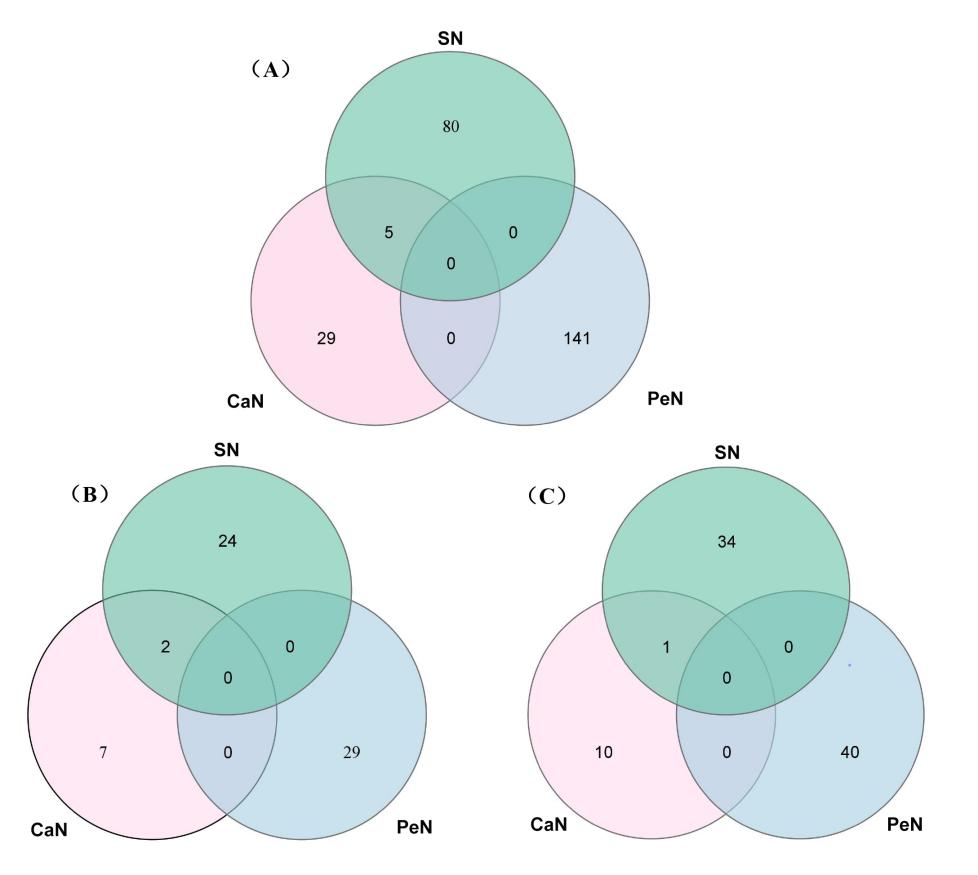


**Fig. S4** Venn diagram. (A)The number of SNPs. (B) GWAS-related *cis*-eQTLs. (C) Genes regulated by GWAS-related *cis*-eQTLs that significantly (*P* < 0.01) associated SNPs with that of CaN, PeN and SN.


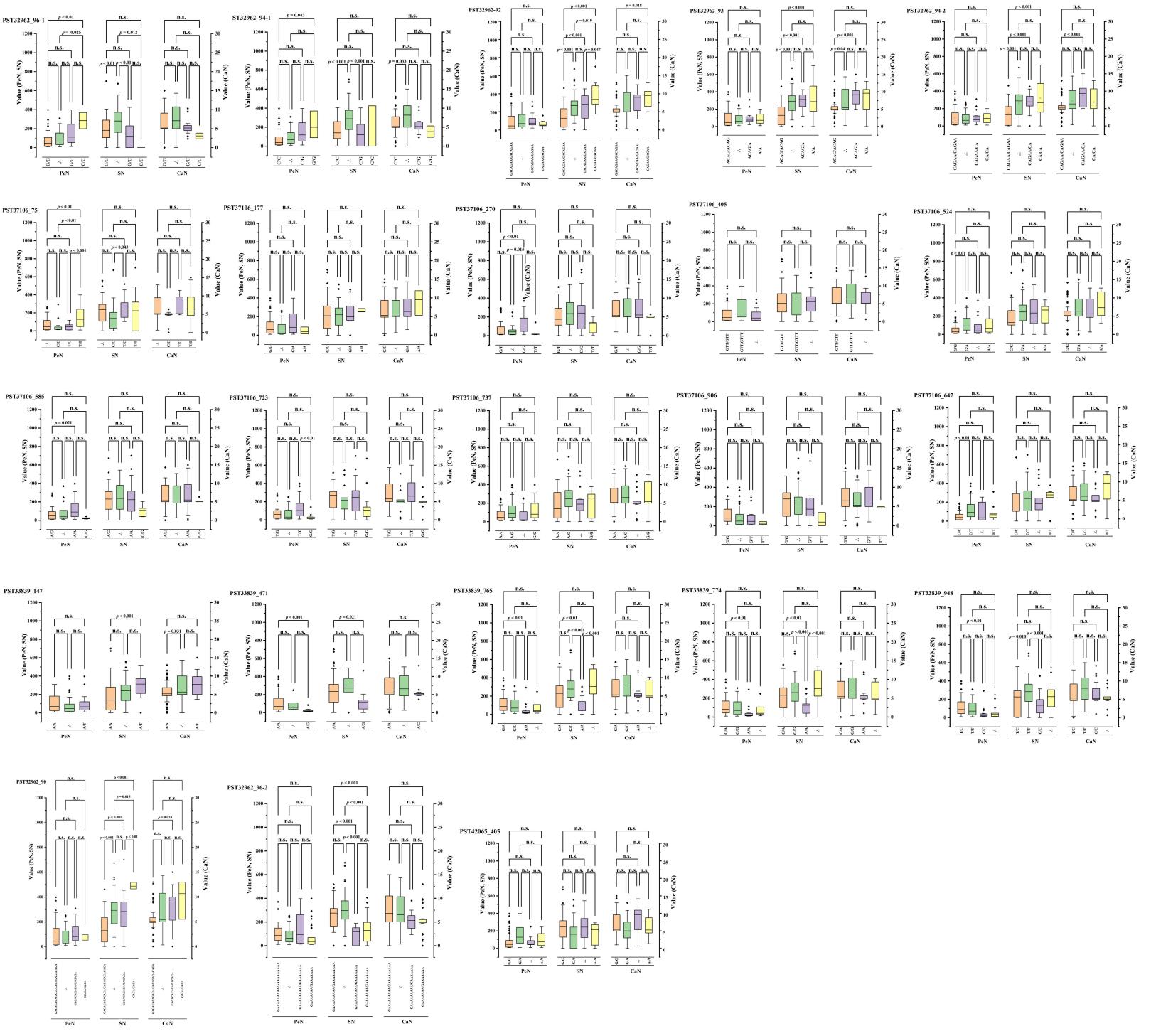


**Fig. S5** The divergence of floral organ number between different alleles of the eQTLs.
